# Exploratory data of the microalgae compounds for food purposes

**DOI:** 10.1016/j.dib.2020.105182

**Published:** 2020-01-25

**Authors:** Tatiele C. do Nascimento, Pricila P. Nass, Andrêssa S. Fernandes, Karem R. Vieira, Roger Wagner, Eduardo Jacob-Lopes, Leila Q. Zepka

**Affiliations:** Department of Food Technology and Science, Federal University of Santa Maria (UFSM), Santa Maria, RS, 97105-900, Brazil

**Keywords:** Microalgae, Carotenoid, Fatty acid, Antioxidant capacity, Compounds bioactive

## Abstract

This brief data article refers to the previous exploration of *Scenedesmus obliquus* and *Phormidium autumnale* biomass about the possibility of using these microalgae species as an unconventional functional food. Data on chemical composition, fatty acids, volatile compounds, and carotenoid profiles were determined. In parallel, are provided the antioxidant capacity (reducing capacity - RC and reactive oxygen species deactivation - ORAC) of aqueous, lipophilic, and carotenoid extracts isolated from microalgae biomass. Both species have similar compounds in their biomass. However, *S. obliquus* was statistically different with a lower saturated fatty acid (STF) followed by higher mono (MUFA) and polyunsaturated (PUFA) content, also showed higher antioxidant potential for acetone extract and isolated carotenoids. On the other hand, *P. autumnale* aqueous extract showed high RC and ORAC. The significance of the experimental data was determined using the *t*-test (p < 0.05) based on the Statistica 7.0 software. These findings led us to explore the microalgae *S. obliquus* in an *in vivo* experimental model.

**Table:** Specifications Table

Subject	*Food Science*
Specific subject area	*Bioactive Compounds From Microalgae*
Type of data	*Table*
How data were acquired	*Microalgae biomass chemical composition has been characterized according to AOAC, 2002; The fatty acid composition was determined by using Agilent capillary gas chromatography system, Series 6850, flame ionization detector (FID); The volatile compounds was obtained by GC-MS/MS; The carotenoids were analyzed by HPLC using a diode array detector (PDA) (model SPD-M20A) and a mass spectrometer with an ion-trap analyzer and atmospheric pressure chemical ionization (APCI) source; Antioxidant capacity obtained from ORAC method by microplate latter.*
Data format	*Raw and Analyzed*
Parameters for data collection	*These are described in the text description of the data*
Description of data collection	*These are described in the text description of the data*
Data source location	*Department of Food Technology and Science, Federal University of Santa Maria (UFSM), P.O. Box 5021, Santa Maria, 97105–900, Brazil*
Data accessibility	*With the article*
Related research article	*Nascimento* et al.*, Microalgae carotenoids intake: influence on cholesterol levels, lipid peroxidation and antioxidant enzymes. Food Res. Int., 108 (2020) 108770*
Subject	*Food Science*
Specific subject area	*Chemistry (General) and food science.*
Type of data	*Table*

**Table:** 

**Value of the Data** • The data provided may be useful for comparing the chemical constitution between microalgae species. • These data extend the knowledge to the database of the quantitative and qualitative profile of biocompounds from microalgae biomass with potential for application as food components. • The data provided is useful for functional food industries seeking natural alternatives as a source of bioactive compounds. • These data present a relevant screening about the antioxidant potential of microalgae biomass, which may contribute to the expansion of the database since this information in the literature is still limited

## 1 Data

Here we report exploratory, experimental data on chemical composition analysis (Table 1), fatty acid profile (Table 2), antioxidant capacity (Table 3), carotenoid profile (Table 4), and volatile organic compounds (Table 5) of two microalgae (*S. obliquus* and *P. autumnale*) to explore as functional food proposals. Among them, *S. obliquus* was more attractive due to its fatty acid content and antioxidant capacity of lipophilic compounds.

**Table 1:** Chemical characterization of microalgae biomass.

Constituent	*P. autumnale*^1^	*S. obliquus*^1^
Lipids	15.49 ± 0.92^a^	15.64 ± 0.08^a^
Proteins	50.20 ± 0.22^a^	50.40 ± 0.17^a^
Moisture	4.01 ± 0.87^a^	5.01 ± 0.35^a^
Minerals	7.12 ± 1.00^a^	5.36 ± 0.51^a^
Fiber	0.72 ± 0.01^a^	0.76 ± 0.02^a^
Carbohydrates	22.43 ± 0.74^a^	22.81 ± 1.00^a^

1 Value (% dry weight). Values (rows) followed by different superscript letters indicate statistical differences (p < 0.05).

**Table 2:** Fatty acid profile of the *P. autumnale* and *S. obliquus* biomass.

Fatty Acids	Relative peak area (%)	
	*P. autumnale*	*S. obliquus*
capric (C10:0)	1.84 ± 0.05	1.27 ± 0.03
lauric (C12:0)	0.82 ± 0.01	0.49 ± 0.00
myristic (C14:0)	1.20 ± 0.01	0.65 ± 0.01
pentadecylic (C15:0)	0.31 ± 0.02	0.21 ± 0.03
palmitic (C16:0)	49.53 ± 0.21	27.27 ± 0.35
palmitoleic (C16:1)	8.45 ± 0.31	13.02 ± 0.06
margaric (C17:0)	1.40 ± 0.06	0.45 ± 0.00
stearic (C18:0)	4.11 ± 0.14	2.38 ± 0.01
oleic (C18:1n9)	1.60 ± 0.02	13.73 ± 0.13
linoleic (C18:2n6)	24.98 ± 0.20	17.47 ± 0.27
α-linolenic (C18:3n3)	3.13 ± 0.23	17.90 ± 0.02
stearidonic (C18:4n3)	0.24 ± 0.20	2.78 ± 0.03
behenic (C22:0)	0.34 ± 0.07	0.43 ± 0.01
lignoceric (C24:0)	2.05 ± 0.02	1.18 ± 0.02

SFA Ʃ	61.60 ± 0.13^a^	34.31 ± 0.36^b^

MUFA Ʃ	10.05 ± 0.40^b^	26.75 ± 0.09^a^

PUFA Ʃ	28.35 ± 0.28^b^	38.16 ± 0.32^a^

Values (rows) followed by different superscript letters indicate statistical differences (p < 0.05).

**Table 3:** Determination of antioxidant capacity from microalgae extracts.

Antioxidant activity	Extracts	*P. autumnale*	*S. obliquus*
RC^1^	Aqueous	161.64 ± 0.02^a^	155.62 ± 0.00^b^
	50% acetone	155.90 ± 0.04^b^	158.85 ± 0.00^a^
	Isolated carotenoids	nd^3^	nd
ORAC-H^2^	Aqueous	46.95 ± 1.86^a^	33.22 ± 0.29^b^
	50% acetone	nd	nd
	Isolated carotenoids	nd	nd
ORAC-L^2^	Aqueous	nd	nd
	50% acetone	61.53 ± 3.84^b^	78.03 ± 6.33^a^
	Isolated carotenoids	641.85 ± 101.25^b^	1779.9 ± 142.83^a^

Values (rows) followed by different superscript letters indicate statistical differences (p < 0.05).

1 mg EAG. g^−1^.

2 μmol TE.g^−1^.

3 Not determined.

**Table 4:** Carotenoids profile of the *P. autumnale* and *S. obliquus.*

Carotenoids	Carotenoid Content (%)		UV–Vis characteristics			Fragment ions (positive mode) *(m/z*)	
	*P. autumnale*	*S. obliquus*	λmáx (nm)^a^	III/II (%)^b^	AB/II (%)^c^	[M+H]^+^	MS/MS
13-*cis*-neoxanthin	0.75 ± 0.02	nd^d^	326, 418, 443, 471	70	35	601	583 [M H − 18]^+^, 565, 509 [M + H − 92]^+^, 491 [M + H − 18 − 92]^+^, 221
all-*trans*-neoxanthin	0.49 ± 0.02	nd	415, 438, 468	78	0	601	583 [M + H − 18]^+^, 565, 509 [M + H-92]^+^, 491 [M + H-18-92]^+^, 221
9-*cis*-neoxanthin	0.73 ± 0.02	2.18 ± 0.21	328, 412, 435, 464	75	22	601	583 [M + H − 18]^+^, 565 [M + H − 18 − 18]^+^, 547 [M + H − 18 − 18 − 18]^+^, 509 [M + H − 92]^+^
all-*trans*-violaxanthin	nd	1.14 ± 0.10	414, 437, 466	56	0	601	583 [M + H − 18]^+^, 565 [M + H − 18 − 18]^+^, 509 [M + H − 92]^+^, 221
all-*trans*-luteoxanthin	nd	1.97 ± 0.03	406, 421, 447	62	0	601	583 [M + H − 18]^+^, 565 [M + H − 18 − 18]^+^, 509 [M + H − 92]^+^, 491 [M + H − 92 − 18]^+^, 221
all-*trans*-antheraxanthin	nd	1.38 ± 0.2	419, 445, 471	50	0	585	567 [M + H − 18]^+^, 549 [M + H − 18 − 18]^+^, 531 [M + H − 18 − 18 − 18]^+^, 493 [M + H − 92]^+^, 221
9-*cis*-violaxanthin	0.92 ± 0.01	nd	329, 419, 440, 465	70	9	601	583 [M + H − 18]^+^, 565 [M + H − 18 − 18]^+^
13-*cis*-lutein	0.44 ± 0.12	nd	330, 416, 437, 464	35	46	569	551, 533, 495, 477, 459
all-*trans*-diatoxanthin	nd	0.76 ± 0.03	425, 449, 472	9	nc^e^	567	549 [M + H − 18]^+^, 535, 531 [M + H − 18 − 18]^+^, 475 [M + H − 92]^+^, 393
all-*trans*-lutein	17.98 ± 0.01	26.92 ± 0.06	420, 444, 472	59	0	569	551 [M + H − 18]+ (in source), 533 [M + H − 18 − 18]^+^, 495 [M + H − 18 − 56]^+^
15-*cis*-zeaxanthin	nd	1.39 ± 0.08	420, 449, 474	16	nc	569	551 [M + H − 18]^+^, 533 [M + H − 18 − 18]^+^, 477 [M + H − 92]^+^
13-*cis*-zeaxanthin	0.02 ± 0.00	nd	334, 421, 440, 471	nc	40	569	551 [M + H − 18]^+^, 533, 495, 477 [M + H − 92]^+^, 459 [M + H − 106]^+^
all-*trans*-zeaxanthin	13.53 ± 0.07	9.46 ± 0.03	425, 450, 476	30	0	569	551 [M + H − 18]^+^, 533 [M + H − 18 − 18]^+^, 477 [M + H − 92]^+^
9-*cis*-lutein	0.43 ± 0.01	1.04 ± 0.05	331, 415, 441, 467	50	11	569	551 [M + H − 18]^+^ (in source), 533 [M + H − 18 − 18]^+^, 495 [M + H − 18 − 56]^+^
9-*cis*-zeaxanthin	0.15 ± 0.01	1.11 ± 0.06	419, 446, 470	33	nc	569	551 [M + H − 18]^+^, 533 [M + H − 18 − 18]^+^, 477 [M + H − 92]^+^
all-*trans*-canthaxanthin	0.26 ± 0.07	0.36 ± 0.02	470/472	0	0	565	547 [M + H − 18]^+^, 509 [M + H − 56]^+^, 459 [M + H − 106]^+^, 363, 203
*cis-*carotenoid	0.24 ± 0.02	nd	330, 416, 444, 468	20	26	555	537
*cis-*carotenoid	0.27 ± 0.01	nd	339, 420, 442, 465	36	21	567	535, 444
*cis-*carotenoid	0.49 ± 0.01	nd	345, 421, 446, 471	30	25	569	551 [M + H − 18]^+^, 533 [M + H − 18 − 18]^+^, 495, 477 [M + H − 92]^+^, 459
5,6-β-carotene-epoxide	nd	0.74 ± 0.02	419, 445, 473	64	0	553	535 [M + H − 18]^+^, 461 [M + H − 92]^+^, 205
all-*trans*-β-cryptoxanthin	nd	0.86 ± 0.02	425, 450, 476	18	0	553	535 [M + H − 18]^+^, 461 [M + H − 92]^+^
all-*trans*-zeinoxanthin	3.61 ± 0.12	nd	420, 448, 473	48	0	553	535 [M + H − 18]^+^, 461 [M + H − 92]^+^, 361
all-*trans*-echinenone	5.05 ± 0.06	6.01 ± 0.12	459/462	0	0	551	533 [M + H − 18]^+^, 427, 203
15-*cis*-β-carotene	0.25 ± 0.02	nd	337, 420, 449, 471	5	50	537	457 [M + H − 80]^+^, 444 [M − 92]^+^, 399 [M − 137]^+^, 177
13-*cis*-β-carotene	nd	1.62 ± 0.07	338, 420, 445, 470	14	48	537	444 [M + H − 92]^+^, 347
*cis*-echinenone	11.06 ± 0.06	3.84 ± 0.16	457/454	0	nc	551	533 [M + H − 18]^+^, 471 [M + H − 80]^+^, 427
all-*trans*-α-carotene	3.81 ± 0.24	1.51 ± 0.01	419, 445, 473	62	0	537	413, 321
all-*trans*-β-carotene	34.49 ± 0.52	28.05 ± 0.18	425, 451, 478	33	0	537	444 [M + H − 92]^+^, 399, 355
9-*cis-*β-carotene	1.78 ± 0.07	4.50 ± 0.01	421, 446, 472	30	nc	537	444 [M + H − 92]^+^

a Sectral fine structure.

b Ratio of the height of the longest wavelength absorption peak (III) and that of the middle absorption peak (II).

c Ratio of the *cis* peak (AB) and the middle absorption peak (II).

d Not detected.

e Not calculated.

**Table 5:** Volatile organic compounds of the microalgae P. autumnale and S. obliquus.

LRI DB-Wax^a^	Compounds	Relative Peak Area (%)^b^	
		*P. autumnale*	*S. Obliquus*
611	acetaldehyde	0.29 ± 0.02	0.24 ± 0.02
626	propanal	0.01 ± 0.00	0.21 ± 0.00
632	2-methyl propanal	nd^c^	0.06 ± 0.01
634	2-propanone	4.77 ± 0.34	0.55 ± 0.05
639	4-methyl-3-pentenal	0.01 ± 0.00	nd
643	2-propenal	nd	0.02 ± 0.00
653	2-methyl furan	0.17 ± 0.01	0.10 ± 0.01
656	butanal	0.12 ± 0.01	0.32 ± 0.02
670	2-butanone	0.89 ± 0.03	0.58 ± 0.02
673	methyl propionate	nd	0.38 ± 0.01
676	2-methyl butanal	0.09 ± 0.00	0.07 ± 0.01
679	3-methyl butanal	0.09 ± 0.00	0.67 ± 0.03
693	2-propanol	0.15 ± 0.00	nd
1018	ethyl propanoate	nd	1.46 ± 0.11
1031	ethyl isobutanoate	nd	0.47 ± 0.03
1047	pentanal	0.72 ± 0.02	2.49 ± 0.18
1086	2,6-dimethyl nonane	0.31 ± 0.00	0.22 ± 0.01
1115	toluene	0.99 ± 0.01	0.78 ± 0.02
1123	propanol	0.15 ± 0.00	5.33 ± 0.24
1124	3-methyl-1-buten-3-ol	0.20 ± 0.01	nd
1129	ethyl 2-methylbutyrate	nd	0.04 ± 0.00
1133	2,3-pentanedione	0.04 ± 0.00	0.16 ± 0.00
1137	2-ethyl-3-methylbutanal	0.01 ± 0.00	0.03 ± 0.00
1146	hexanal	3.90 ± 0.23	3.16 ± 0.15
1149	methyl pentanoate	nd	0.29 ± 0.03
1170	3-pentanol	nd	0.04 ± 0.00
1178	2-nonanol	0.07 ± 0.01	nd
1179	2-pentenal	nd	1.64 ± 0.08
1190	2-ethyl-*trans*-2-butenal	nd	0.18 ± 0.02
1193	butanol	0.98 ± 0.03	3.02 ± 0.08
1215	2-nonanone	0.04 ± 0.00	nd
1230	limonene	0.43 ± 0.02	0.32 ± 0.01
1233	3-penten-2-ol	nd	0.12 ± 0.01
1246	1,8-cineole	0.12 ± 0.00	0.14 ± 0.01
1251	3-methyl butanol	0.84 ± 0.07	8.01 ± 0.69
1258	2-hexenal	nd	3.31 ± 0.20
1266	2-pentyl furan	0.63 ± 0.03	0.03 ± 0.00
1274	ethyl hexanoate	0.09 ± 0.00	1.52 ± 0.11
1278	6-methyl-2-heptanone	0.47 ± 0.04	nd
1294	1-pentanol	3.37 ± 0.22	4.28 ± 0.18
1325	3-penten-1-ol	nd	0.20 ± 0.02
1330	octanal	0.31 ± 0.02	0.17 ± 0.02
1362	2-butyl octanol	nd	2.46 ± 0.17
1363	2-propyl heptanol	4.46 ± 0.28	4.50 ± 0.20
1385	6-methyl-hept-5-en-2-one	2.22 ± 0.04	0.61 ± 0.02
1407	hexanol	11.77 ± 0.59	11.05 ± 0.24
1461	3-hexen-1-ol	nd	0.03 ± 0.00
1473	nonanal	0.41 ± 0.02	nd
1500	2-hexen-1-ol	nd	0.88 ± 0.04
1529	1-octen-3-ol	1.15 ± 0.02	1.76 ± 0.20
1535	heptanol	1.24 ± 0.15	0.89 ± 0.05
1543	2-cyclohexen-1-one	0.14 ± 0.01	0.39 ± 0.02
1558	2-ethyl hexanol	4.36 ± 0.27	nd
1577	2-ethyl-2-pentenal	nd	0.36 ± 0.05
1586	n-tridecanol	0.34 ± 0.04	nd
1596	linalool	0.28 ± 0.01	nd
1606	octanol	0.99 ± 0.10	nd
1621	3,5-octadien-2-one	0.18 ± 0.00	0.73 ± 0.01
1647	β-caryophyllene	nd	0.67 ± 0.07
1658	oct-3-en-2-ol	35.68 ± 0.78	18.42 ± 1.29
1663	nonadecanol	0.43 ± 0.02	nd
1671	β-cyclocitral	5.77 ± 0.26	1.15 ± 0.14
1682	butyrolactone	0.05 ± 0.01	1.91 ± 0.10
1695	safranal	0.66 ± 0.03	1.11 ± 0.06
1707	nonanol	1.53 ± 0.05	nd
1702	1,4-cyclohexanedione	nd	0.19 ± 0.02
1715	3-ethyl-2,4-pentanedione	0.83 ± 0.02	nd
1724	γ-valerolactone	0.17 ± 0.00	0.47 ± 0.05
1747	keto-Isophorone	nd	2.71 ± 0.35
1759	γ-hexalactone	nd	2.64 ± 0.31
1784	tetradecanol	nd	0.55 ± 0.07
1786	l-carvone	nd	0.07 ± 0.01
1835	3,4-dimethylcyclohexanol	0.86 ± 0.04	0.14 ± 0.01
1855	2,5-dimethyl-1-hepten-4-ol	0.05 ± 0.00	0.10 ± 0.00
1855	2-ethyl butanal	nd	0.13 ± 0.00
1869	γ-heptalactone	0.02 ± 0.00	0.13 ± 0.01
1892	furan	0.10 ± 0.01	0.05 ± 0.00
1889	α-ionone	nd	1.42 ± 0.20
1918	4,8-dimethyl-1,7-nonadien-4-ol	1.14 ± 0.01	1.86 ± 0.21
1988	*trans*-β-ionone	3.83 ± 0.32	2.09 ± 0.28
2000	benzothiazole	0.12 ± 0.01	0.18 ± 0.02
2002	6-methyl-7-octen-2-one	0.04 ± 0.01	0.61 ± 0.01
2006	dodecanol	0.09 ± 0.00	0.07 ± 0.01
2028	7,8-epoxy-α-ionone	nd	0.08 ± 0.00
2038	phenol	0.03 ± 0.00	0.17 ± 0.02
2044	β-ionone epoxide	0.92 ± 0.03	1.17 ± 0.15

a Linear Retention Indices in the DB-Wax column.

b Mean and standard deviation often independent experiments.

c nd: not detected.

## 2 Experimental design materials and methods

### 2.1 Microalgae and culture media

Axenic cultures of *Scenedesmus obliquus* (CPCC05) were obtained from the Canadian Phycological Culture Centre. Axenic cultures of *Phormidium autumnale* were initially isolated from the Cuatro Cienegas desert, in Mexico (26°59′ N, 102°03′ W). Stock cultures were propagated in solidified agar-agar (20 g L^−1^) containing synthetic BG11 medium [1]. The incubation conditions used were 25 °C, the light intensity was constant 30 μmol m^−2^ s^−1^, and a photoperiod of 12 h.

### 2.2 Microalgae biomass production

The biomass production was carried according to Deprá et al. [2], where details of reactor configuration, operational conditions, and downstream processing were described. The biomass was separated from the culture medium by centrifugation (10000 rpm, 10 min, 10 °C), the supernatant was discarded, and the remaining biomass was freezing at −18 °C for 24 hours. After, the biomass was freeze-dried for 24 h at −50 °C above −175 μm Hg and then stored at −18 °C until analysis.

### 2.3 Chemical composition

Microalgae biomass chemical composition has been characterized according to AOAC [3]. Carbohydrate content has been estimated by difference [Carbohydrate% = 100% - (proteins % + lipids % + minerals % + fibers %)].

### 2.4 Fatty acids profile

The method of Hartman and Lago [4] was used to obtain the dried lipid extract and later the fatty acid methyl esters (FAMEs). The fatty acid composition was determined by using Agilent capillary gas chromatography system, Series 6850, flame ionization detector (FID) (Agilent, Santa Clara-CA, USA), with an Agilent DB-23 capillary column (50% cyanopropyl-methylpolysiloxane; length 60 m, internal diameter 0.25 mm and 0.25 μm film thickness). The FAMEs were identified by comparison of the retention times with the authentic standards from FAME Mix C4–C24 (18919-1AMP, Supelco Sigma-Aldrich, St. Louis-MI, USA). The quantification was based on relative peak areas.

### 2.5 Extracts of microalgae biomass

The aqueous and 50% acetone extracts were obtained according to the adaptations of Shanab et al. [5] and Ou et al. [6], respectively. The lyophilized biomass (0.5 ± 0.01 g) was dissolved in 10 mL water and 50% acetone for the obtention of the two extracts. Both extracts were agitated for 1 hour, protected from light exposure. They were then centrifuged for 15 min at 1400 rpm at 25 °C, and the supernatant was separated. This procedure was repeated two times. The extract was stored under an N_2_ atmosphere and kept at −80 °C until the antioxidant screening.

### 2.6 Carotenoids profile

The carotenoids were determinate, according to Rodrigues et al. [7]. The freeze-dried biomass (0.1 ± 0.02 g) were exhaustively extracted with ethyl acetate and methanol in a mortar with a pestle followed by centrifugation (Hitachi, Tokyo, Japan) for 7 min at 1500×*g*. The exhaustion was obtained from 9 to 5 extractions with 10 mL of ethyl acetate and MeOH, respectively. The time per extraction was approximately 5 minutes. The homogenized sample suspension was filtered through a 0.22 μm polyethylene membrane, concentrated in a rotary evaporator (T < 30 °C), suspended in a mixture of petroleum ether/diethyl ether [1:1 (v/v)], and saponified for 16 h with 10% (w/v) methanolic KOH at room temperature. The alkali was removed by washing with distilled water, and the extract was once again concentrated in a rotary evaporator, was placed in the N_2_ atmosphere, and kept at −37 °C in the dark until chromatographic analysis. The carotenoids were analyzed by HPLC (Shimadzu, Kyoto, Japan) using a diode array detector (PDA) (model SPD-M20A) and a mass spectrometer with an ion-trap analyzer and atmospheric pressure chemical ionization (APCI) source (model Esquire 4000, Bruker Daltonics, Bremem, Germany) [8]. The carotenoid separation was performed on a C30 YMC column (5 μm, 250 × 4.6 mm) (Waters, Wilmington-DE, USA). HPLC-PDA-MS/MS parameters were: mobile phase constituted of the mixture of MeOH and MTBE, a linear gradient of 95:5 to 70:30 in 30 min, to 50:50 in 20 min underflow rate was 0.9 mL.min^−1^. The identification was according to the following combined information: elution order on C30 HPLC column, co-chromatography with authentic standards, UV–Visible spectrum, mass spectral characteristics, and comparison with literature data. The carotenoids were quantified by HPLC-PDA, using five-point analytical curves.

### 2.7 Antioxidant capacity of biomass and carotenoid extract

#### 2.7.1 ORAC assay

The antioxidant capacity of the microalgae biomass was carried out according to the oxygen radical absorbance capacity method (ORAC) [6]. For the aqueous extract, the reaction medium was phosphate buffer, while for a lipophilic extract from biomass and carotenoid extract, 7% of randomly methylated beta-cyclodextrin (RMCD) in 50% acetone solution was added. The fluorescence signal was recorded every 1 min–160 min on the Biotek Microplate Reader (Biotek. Winooski-VT, USA) with Gen5™ 2.0 data analysis software using 520 nm emission wavelength and 485 nm excitation. Results were expressed as μmol equivalent of Trolox by dry weight microalgae biomass.

#### 2.7.2 Reduction capacity

The reducing capacity of the extracts (aqueous and 50% acetone) was measured by your ability to reduce Folin-Ciocalteu reagent. The Folin-Ciocalteu method was adapted to Singleton and Rossi [9], 2.5 mL of diluted samples were added to 0.5 mL of 1:10 diluted Folin-Ciocalteu reagent. After 5 min, 2 mL of 7.5% sodium carbonate was added. After two h of incubation at room temperature, the absorbance at 760 nm was measured. Gallic acid (11–70 μg mL^−1^) was used for the standard calibration curve. The results were expressed as gallic acid equivalent per gram dry weight of microalgae (mg GAE. g^−1^).

### 2.8 Extraction, identification and quantification of volatile compounds

#### 2.8.1 Isolation of the volatile organic compounds

The volatile compounds were isolated from the matrix using headspace solid-phase microextraction (HS-SPME) divinylbenzene/Carboxen/polydimethylsiloxane (DVB/Car/PDMS) fiber (50/30 μm film thickness × 20 mm; Supelco, Bellefonte, PA) for gas chromatography-mass spectrometry (GC-MS) analysis [10]. A 0.2 ± 0.02 g aliquot of the microalgae biomass was added to a 20 mL screw-top vial with hole cap PTFE/silicone septum (Supelco, Bellafonte, PA). The SPME fiber was exposed to the headspace of the vial for 60 min at 40 °C. After this period, the fiber was removed from the vial and submitted to chromatographic analysis [11].

#### 2.8.2 GC/MS analysis

The volatile compounds were analyzed according to Santos et al. [10] by Shimadzu QP 2010 Plus gas chromatography coupled to the mass spectrometer (Shimadzu, Kyoto, Japan). Thus, the fiber was thermally desorbed for 15 min in the split/splitless injector, operating in splitless mode (1.0 min splitter off) at 250 °C. Helium was used as a carrier gas at constant 1.6 mL.min^−1^. The analytes were separated on a DB-Wax fused silica capillary column, 60 m in length, 0.25 mm id, and 0.25 μm film thickness (Chrompack Wax 52-CB). The initial column temperature was set at 35 °C for 5 min, followed by a linear increase at 5 °C.min^−1^ to 220 °C, and this temperature was held for 5 min. The MS detector was operated in electron impact ionization mode +70 eV, and mass spectra obtained by a scan range from *m*/*z* 35 to 350 [10]. The volatile compounds were identified by a comparison of experimental MS spectra with those provided by the computerized library (NIST MS Search). Also, the linear retention index (LRI) was calculated for each volatile compound using the retention times of a standard mixture of paraffin homologs series (C6–C24) to aid the identification [12]. Analytes were quantified based on relative peak areas.

### 2.9 Statistical analysis

The analysis was performed using Statistica 7.0 software (Statsoft, Tulsa-OK, USA). The significance of the experimental data was determined using a *t*-test (*p* < 0.05).
